# Psychostimulant treatment uniquely reduces left uncinate fasciculus microstructural integrity in ADHD youth with a familial risk for bipolar I disorder: a 12‐week DTI study

**DOI:** 10.1111/jcpp.70053

**Published:** 2025-09-17

**Authors:** Kun Qin, Wen Chen, Du Lei, Ziyu Zhu, Maxwell J. Tallman, Nanfang Pan, Lisha Zhang, Luis Rodrigo Patino, John A. Sweeney, Melissa P. DelBello, Robert K. McNamara

**Affiliations:** ^1^ Department of Radiology Taihe Hospital, Hubei University of Medicine Shiyan China; ^2^ Department of Psychiatry and Behavioral Neuroscience University of Cincinnati College of Medicine Cincinnati OH USA; ^3^ Mental Health Center Taihe Hospital, Hubei University of Medicine Shiyan China; ^4^ School of Medical Informatics Chongqing Medical University Chongqing China; ^5^ Huaxi MR Research Center (HMRRC), Department of Radiology, The Center for Medical Imaging West China Hospital of Sichuan University Chengdu China; ^6^ The Turner Institute for Brain and Mental Health, School of Psychological Sciences and Monash Biomedical Imaging Monash University Clayton Australia

**Keywords:** Attention‐deficit/hyperactivity disorder, bipolar I disorder, familial risk, stimulants, adolescent

## Abstract

**Background:**

Bipolar I disorder (BD) is associated with reduced white matter microstructural integrity in the uncinate fasciculus (UF), a primary fiber tract connecting frontolimbic systems. Although familial history for BD, attention‐deficit/hyperactivity disorder (ADHD), and psychostimulants are important risk factors implicated in BD pathoetiology, their impact on UF microstructure remains poorly understood.

**Methods:**

This diffusion tensor imaging study investigated UF microstructural integrity prior to and following 12 weeks of psychostimulant treatment in ADHD youth with (‘high‐risk’, HR) and without (‘low‐risk’, LR) a first‐degree relative with BD. Healthy controls were included for comparative purposes. LR youth received 12‐week open‐label mixed amphetamine salts‐extended release (MAS‐XR), and HR youth were randomized to either MAS‐XR or placebo (PLA). Bilateral UF fractional anisotropy (FA) and axial diffusivity (AD) were assessed using automated fiber quantification.

**Results:**

A total of 137 participants were included in the analyses. At baseline, there were no significant group differences in bilateral UF microstructural metrics. Following 12‐week MAS‐XR treatment, significant group‐by‐time interactions were found for left UF FA and AD between HR‐MAS and LR‐MAS, as well as for left UF FA between HR‐MAS and HR‐PLA. Specifically, left UF FA and UF AD decreased significantly in HR‐MAS but remained unchanged in LR‐MAS and HR‐PLA groups. At week 12, left UF FA was lower in HR‐MAS relative to HC but not in LR‐MAS or HR‐PLA. Segment‐wise analyses further revealed that UF microstructural changes in the HR‐MAS group were localized to the anterior segments.

**Conclusions:**

These results suggest that HR‐ADHD youth are uniquely vulnerable to reductions in left UF microstructural integrity following psychostimulant treatment, suggesting potential relevance to BD pathoprogression.

## Introduction

The initial onset of mania, and by definition bipolar I disorder (BD), most frequently occurs during adolescence (Perlis et al., 2009), a developmental period involving progressive structural and functional changes in frontolimbic circuitry (Gerber et al., 2009; Gogtay et al., 2004). Diffusion tensor imaging (DTI) studies have shown that this period is associated with a progressive increase in white matter microstructural integrity in limbic forebrain fiber tracts including the uncinate fasciculus (UF) (Barnea‐Goraly et al., 2005; Hasan et al., 2009; Schmithorst, Wilke, Dardzinski, & Holland, 2002). The UF is the principal white matter tract connecting the ventral and orbital prefrontal cortex with anterior temporal lobe structures including the amygdala (Von Der Heide, Skipper, Klobusicky, & Olson, 2013). Age‐related increases in UF fractional anisotropy (FA) are associated with dynamic changes in prefrontal–amygdala functional connectivity and reductions in amygdala reactivity to emotional stimuli (Gee et al., 2013; Goetschius et al., 2019; Hein et al., 2018; Wu et al., 2016). Consistent with a disruption in UF maturation, subjects with BD exhibit prefrontal–amygdala functional dysregulation and bilateral reductions in UF FA compared with healthy controls (Mesbah et al., 2023; Xu et al., 2022). Moreover, prospective studies have found that youth with BD do not exhibit the normal age‐related increase in UF FA (Weathers et al., 2018) and that lower UF FA, but not other major white matter tracts, is a significant predictor of BD in offspring of parents with BD (Li et al., 2021). While these findings suggest that deficits in UF microstructural integrity are relevant to the pathoetiology of BD, contributing risk mechanisms remain poorly understood.

Having a first‐degree relative with BD robustly increases the risk for developing BD (Mortensen, Pedersen, Melbye, Mors, & Ewald, 2003), as well as other psychiatric disorders including attention‐deficit/hyperactivity disorder (ADHD) (Khoury, Acquaviva, Purper‐Ouakil, Delorme, & Ellul, 2023; Lau et al., 2018; Propper et al., 2023). Indeed, prevalence rates of ADHD in youth with a BD family history are significantly higher than youth without a BD family history (Birmaher et al., 2021; Khoury et al., 2023). Importantly, ADHD commonly precedes the initial onset of BD (Axelson et al., 2015; Meier et al., 2018), and ADHD significantly increases the risk for developing BD (Brancati, Perugi, Milone, Masi, & Sesso, 2021). Unlike youth that developed BD, however, cross‐sectional DTI evidence indicates that UF FA is either not altered (Ashtari et al., 2005; Hamilton et al., 2008; Lawrence et al., 2013), or is increased (Konrad et al., 2010; Peterson et al., 2011; Silk, Vance, Rinehart, Bradshaw, & Cunnington, 2009; Tamm, Barnea‐Goraly, & Reiss, 2012), in ADHD youth compared with healthy youth despite lower FA in other major fiber tracts including the corpus callosum (Chen et al., 2016). However, the latter ADHD studies did not control for BD family history, and UF microstructural integrity has never been systematically investigated in ADHD youth with a BD family history.

Psychostimulants are currently the first‐line treatment for ADHD regardless of BD family history. Although controversial, some (DelBello et al., 2001; Faedda, Baldessarini, Glovinsky, & Austin, 2004; Jerrell, McIntyre, & Park, 2014; Soutullo et al., 2002), but not all (Tillman & Geller, 2006), evidence suggests that antecedent psychostimulant exposure can precipitate mood symptoms and accelerate the onset of mania in a subset of vulnerable ADHD youth. However, the role of BD family history and the central mechanisms conferring vulnerability in ADHD youth are not known. Although there is currently limited information regarding the effects of psychostimulant medications on UF microstructural integrity, a cross‐sectional study did not observe differences in UF FA in stimulant‐naïve and stimulant‐treated ADHD youth (de Luis‐García et al., 2015), and cumulative psychostimulant exposure was not associated with UF FA in ADHD youth (Schweren, de Zeeuw, & Durston, 2013). A controlled trial in initially stimulant‐naïve ADHD boys found that 16‐week methylphenidate treatment significantly increased FA in whole brain and multiple fiber tracts (Bouziane et al., 2019). However, the effect of psychostimulant treatment on UF microstructural integrity in ADHD youth with a BD family history has never been systematically investigated.

In the context of a larger treatment trial, this secondary DTI analysis evaluated the effects of 12‐week treatment with mixed amphetamine salts‐extended release (Adderall XR, MAS) on UF microstructural integrity in initially psychostimulant‐free ADHD youth with and without a first‐degree relative with BD. A healthy control (HC) group was also included for comparative purposes and did not receive MAS. Bilateral UF FA and axial diffusivity (AD) were assessed. Based on the evidence reviewed above, we hypothesized that HR‐ADHD youth, but not LR‐ADHD youth, would exhibit UF FA deficits at baseline compared with both HC and LR‐ADHD youth and that HR‐ADHD youth, but not LR‐ADHD youth, would exhibit reductions in UF FA following 12‐week MAS treatment. Exploratory analyses investigated associations among UF microstructural alterations with changes in clinical ratings.

## Methods

### Participants

Three groups of psychostimulant‐free youth (10–18 years) were enrolled: (a) youth with ADHD and at least one biological parent or sibling with BD (‘high‐risk’, HR), (b) youth with ADHD and no first‐ or second‐degree relative with a mood or psychotic disorder (‘low‐risk’, LR), and (c) typically developing HC with no personal or family history of a DSM‐5 Axis I psychiatric disorder. The Structured Clinical Interview for DSM‐5 (SCID‐5‐CV) was administered to confirm a parental or sibling (≥18 years) diagnosis of BD, and the Kiddie Schedule for Affective Disorders and Schizophrenia—Present and Lifetime Version (KSADS‐PL) was administered to confirm a sibling (<18 years) diagnosis of BD. The Family Interview for Genetics Studies (FIGS) was used to confirm DSM‐5 BD diagnoses in other first‐ or second‐degree relatives. HC had no lifetime DSM‐5 Axis I disorder as determined by the KSADS‐PL and had no first‐ or second‐degree relatives with a mood or psychotic disorder as determined by the FIGS.

Participants were recruited from the Cincinnati Children's Hospital Medical Center, the University of Cincinnati Medical Center, and the local community. All participants did not have any contraindication to an MRI scan, had an IQ ≥ 80 as determined by the Wechsler Abbreviated Scale of Intelligence (WASI), had no major medical or neurological illness that could influence MRI results or any significant episode (>10 min) of loss of consciousness, and had no lifetime DSM‐5 substance use disorder. All ADHD youth had no current DSM‐5 mood, conduct, eating or psychotic disorders, Tourette's disorder, chronic tic disorder, or autism spectrum disorder, had no exposure to psychostimulants (prescription or recreational) or other medications used for the treatment of ADHD (e.g., atomoxetine, alpha‐2 agonists) for at least 3 months prior to screening, had no lifetime exposure to mood‐stabilizer or antipsychotic medications, and had no clinically significant electrocardiogram or blood pressure abnormalities. Written informed assent and consent were obtained from youth and their legal guardians, respectively. This study was approved by the University of Cincinnati Institutional Review Board and registered at ClinicalTrials.gov (NCT02478788) June 2015. Enrollment took place from October 2015 to December 2021, and the DTI analyses were initiated in May 2022.

### Treatments

ADHD youth who completed initial screening and the baseline DTI scan began their 12‐week treatment with MAS‐XR. LR‐ADHD (LR‐MAS) youth received open‐label MAS‐XR, and HR‐ADHD youth were randomized (stratified by sex) to double‐blind treatment with MAS‐XR (HR‐MAS) or placebo (HR‐PLA) (Figure S1). MAS‐XR and identical placebo capsules were dispensed by an on‐site investigational pharmacist who assigned treatment according to a randomization schedule generated by the biostatistician. Additionally, a subgroup of HR youth (*n* = 10) who received open‐label MAS‐XR treatment was included for the current analysis. Their symptom responses did not differ significantly from HR youth who received double‐blind MAS‐XR treatment, and excluding these *n* = 10 subjects did not significantly alter the results. In accordance with current MAS‐XR dosage and administration guidelines, the starting dose was 10 mg/day and was increased based on effectiveness and tolerability to within a range of 10 to 40 mg/day. The MAS dose was increased within a range of 10 to 30 mg/day in subjects who were 10 to 12 years old. Medication adherence was monitored by subject interview, pill counts, legal guardian interviews, and a medication adherence diary. LR‐MAS and HR‐MAS groups did not differ in MAS‐XR endpoint dose (LR‐MAS: 19.7 ± 6.7 vs. HR‐MAS: 19.5 ± 6.5, *p* = .93), median dose (LR‐MAS: 17.7 ± 5.2 vs. HR‐MAS: 15.8 ± 5.7, *p* = .12), or mean maximum dose (LR‐MAS: 20.3 ± 6.6 vs. HR‐MAS: 20.9 ± 5.7, *p* = .65), and the group‐by‐time interaction effect was not significant (*p* = .74). All ADHD youth were instructed not to take their daily MAS dose on the day of the endpoint MRI scan (i.e., 24 h washout period) to obviate acute effects.

### Symptom ratings

ADHD symptom ratings were obtained using the clinician‐administered Attention‐Deficit Hyperactivity Disorder Rating Scale (ADHD‐RS), and inattention and hyperactivity/impulsivity subscale scores were analyzed separately. Depression symptom severity was determined using the Children's Depression Rating Scale‐Revised (CDRS‐R), and manic symptom severity with the Young Mania Rating Scale (YMRS). All clinician ratings were administered by two blinded child and adolescent psychiatrists with established inter‐rater reliabilities (kappa > 0.9). Parents completed the Child Behavior Checklist (CBCL), and Dysregulation Profile subscale scores were analyzed.

### DTI data acquisition

MRI data were collected using a Philips Ingenia 3T MRI scanner with a 32‐channel head coil at baseline and week 12. Diffusion‐weighted image acquisition involved a single‐shot, spin‐echo planar imaging sequence in contiguous axial planes that covered the whole brain. Diffusion‐sensitizing gradients were applied in 61 non‐collinear directions, together with seven b0 images. The imaging parameters were as follows: TR = 7,277 ms, TE = 94 ms, matrix = 112 × 112, *b*‐value = 1,000 s/mm^2^, slice thickness = 2 mm, and 60 axial slices. Voxel resolution was 2 × 2 × 2 mm^3^. The subjects were told not to move during the scans, and a dedicated foam filler was used to reduce head movement. After the scan, subjects with brain lesions or obvious artifacts identified in brain scans via visual inspections were excluded from the analyses. To minimize motion‐related artifacts in our analyses, we excluded participants showing excessive head motion defined as either (a) translation greater than 3 mm or rotation greater than 3° in any direction (*X*, *Y*, or *Z* axes), or (b) mean framewise displacements greater than 2 mm. Following these quality control procedures, we further examined potential group‐by‐time interaction effects on all head motion parameters.

### DTI preprocessing and AFQ

Diffusion‐weighted images were preprocessed in native space using the FSL v6.0.0 software (http://www.fmrib.ox.ac.uk/fsl). Briefly, non‐brain tissues were first removed by using the built‐in brain extraction tool in the FSL. Raw images were then corrected for eddy current and head motion distortions using the average of the initial seven non‐diffusion‐weighted *b*0 volumes as a reference. Although head motion correction was applied, residual subtle movements may still influence results (Aoki, Cortese, & Castellanos, 2018). We therefore verified the robustness of our findings by including framewise displacement as an additional covariate in all statistical models. Finally, a diffusion tensor model was fitted at each voxel to estimate whole‐brain maps of diffusion metrics including FA, MD, AD, and RD. We further used the AFQ toolbox to identify the bilateral UF for each participant (Yeatman, Dougherty, Myall, Wandell, & Feldman, 2012; Yeatman, Richie‐Halford, Smith, Keshavan, & Rokem, 2018). The workflow of AFQ can be summarized as six major steps, including (1) whole‐brain deterministic tractography within a white matter mask; (2) segmentation of fiber tracts using waypoint template‐defined regions of interest; (3) refinement of fiber tracts based on the JHU white matter tractography atlas; (4) determination of the core of the fiber tract; (5) fiber tract cleaning by outlier removal; and (6) estimation of diffusion metrics along the fiber tract. Finally, 20 major fiber tracts were identified, and the bilateral UF was selected for analysis according to our study hypothesis. All reconstructed UF tracts were independently evaluated by two neuroradiologists (K.Q. and W.C., who had 5 and 20 years of DTI experience, respectively) via visual inspection based on the following criteria: anatomical plausibility, absence of aberrant streamlines, and terminating patterns consistent with established UF anatomy. Assessments were conducted using directionally encoded color maps co‐registered to T1‐weighted images. Discrepancies were resolved through consensus discussion. DTI scans with UF reconstruction errors would be reprocessed and re‐evaluated prior to final exclusion. Segment‐wise FA and AD were calculated at 100 equidistant segments along the fiber tract. Both mean and segment‐wise diffusion metrics of the bilateral UF were used for the present analyses.

### LR‐MAS vs. HR‐MAS

A linear mixed effects (LME) model was employed to investigate baseline‐endpoint changes in UF microstructural metrics between LR‐MAS and HR‐MAS groups. We used the ‘lmer’ function from the ‘lme4’ package in R (v4.4.1) to fit our linear mixed effects model. Our model included fixed effects for time, group, their interaction, and covariates (age and sex). To account for individual variability, we specified a random intercept for each participant (1|participant) with an unstructured covariance structure. To address potential confounding effects of pubertal status (Genc et al., 2017), we additionally conducted sensitivity analyses by including Tanner stage as an additional covariate in all models. Mean diffusion metrics of the bilateral UF were first examined, followed by fitting the LME model at each of the 100 segments along the fiber tracts. To control for multiple comparisons, we performed False Discovery Rate (FDR) correction using the Benjamini–Hochberg (BH) method with a significance level of adjusted *p* < .05. For metrics exhibiting significant group‐by‐time interaction effects, post hoc simple effect analyses were performed to examine between‐group differences at each time point and within‐group longitudinal changes for each group. These analyses were conducted using estimated marginal means derived from our models, which appropriately account for the model's covariance structure and provide adjusted means. All pairwise comparisons were corrected for multiple testing using the Bonferroni method. Effect size for each significant finding was estimated using Cohen's *d*.

In addition, exploratory correlation analyses among changes in significant UF diffusion metrics and clinical ratings were performed. The correlation was first assessed for LR‐MAS and HR‐MAS groups separately, and the group‐by‐score interaction was examined to determine whether the correlation patterns differed significantly between the two groups. The same BH‐FDR method was implemented to correct for multiple correlations (adjusted *p* < .05).

### Secondary contrasts

Secondary contrasts of interest mainly included comparisons with typically developing healthy controls (HC) and HR placebo controls. Specifically, the following group‐by‐time interactions were investigated: (a) HR‐MAS vs. HC; (b) LR‐MAS vs. HC; (c) HR‐MAS vs. HR‐PLA. The first two compared patient groups with HC to assess the UF microstructural deviations from typically developing levels. The third analysis evaluated psychostimulant treatment effects within the HR‐ADHD group. The LME models controlling for age and sex were used, and the same BH‐FDR correction was applied (adjusted *p* < .05).

### Exploratory whole‐brain analyses

To investigate microstructural changes in other major fiber tracts in addition to the UF, we assessed group‐by‐time interaction effects between the LR‐MAS and HR‐MAS groups for the remaining 18 fiber tracts reconstructed by AFQ. These included bilateral anterior thalamic radiation, corticospinal tract, cingulum cingulate, cingulum hippocampus, inferior fronto‐occipital fasciculus, inferior longitudinal fasciculus, superior longitudinal fasciculus, arcuate fasciculus, as well as splenium and genus of the corpus callosum. The bilateral cingulum hippocampus and right arcuate fasciculus were excluded from the analysis due to failed reconstruction. Additionally, we used Tract‐Based Spatial Statistic (TBSS) to identify white matter microstructural changes at the voxel level (Smith et al., 2006) (see Supporting Information for more details).

## Results

### Demographic and clinical characteristics

A total of 137 youth were included in the analysis (see CONSORT diagrams: Figure S2A,B). There were no significant group differences in age, sex, handedness, or body mass index (Table 1). The ADHD groups did not differ in prior psychostimulant exposure. The LR‐MAS group had a higher percentage of ADHD‐inattention subtype, while the HR‐MAS and HR‐PLA groups had more subjects with ADHD‐combined subtype (*p* = .047). For clinical ratings, at baseline there were no significant group differences in CDRS‐R total score, ADHD‐RS total score, ADHD‐RS inattention, and hyperactivity/impulsivity subscale scores. The HR‐MAS and HR‐PLA groups exhibited significantly higher YMRS (*p* = .015) and CBCL total scores (*p* = .010), as well as CBCL internalizing (*p* = .029) and externalizing (*p* = .017) subscale scores relative to the LR‐MAS group.

**Table 1:** Participant demographic and clinical characteristics

Variables	LR‐MAS (*n* = 47)	HR‐MAS (*n* = 29)	HR‐PLA (*n* = 16)	*p*‐Value	HC (*n* = 45)	*p*‐Value
Age, years	14.15 ± 2.56	14.09 ± 2.51	13.31 ± 2.66	.512	14.70 ± 2.47	.289
Sex, female *N* (%)	16 (34.0%)	11 (37.9%)	4 (25%)	.678	19 (42.2%)	.638
BMI	23.67 ± 6.42	23.71 ± 8.38	24.48 ± 4.72	.914	22.45 ± .4.55	.637
Handedness						
Right	37 (78.7%)	26 (89.7%)	12 (75.0%)	.527	43	.145
Left	7 (14.9%)	1 (3.4%)	2 (12.5%)		2	
Ambidextrous	3 (6.4%)	2 (6.9%)	2 (12.5%)		0	
ADHD subtype, *N* (%)				.047	–	–
ADHD‐C	20 (42.6%)	19 (65.5%)	12 (75.0%)		–	–
ADHD‐I	27 (57.4%)	9 (31.0%)	4 (25.0%)		–	–
ADHD‐H	0 (0.0%)	1 (3.4%)	0 (0.0%)		–	–
Previous psychostimulant use, *N* (%)	24 (51.1%)	22 (75.9%)	12 (75.0%)	.052	–	–
CGI‐S	4.04 ± 0.55	4.28 ± 0.65	4.25 ± 0.77	.231	–	–
ADHD‐RS	33.17 ± 9.83	34.97 ± 9.51	35.56 ± 12.99	.638	–	–
Inattention	20.83 ± 4.83	19.59 ± 5.14	19.19 ± 6.75	.445	–	–
Hyperactivity/Impulsivity	12.34 ± 7.94	15.38 ± 7.22	16.38 ± 7.71	.103	–	–
CDRS‐R	24.06 ± 5.96	27.72 ± 8.59	27.19 ± 8.83	.083	–	–
YMRS	3.06 ± 3.32	5.31 ± 4.20	6.75 ± 8.09	.015	–	–
CBCL total	36.60 ± 17.22	52.67 ± 24.72	50.80 ± 33.16	.010	–	–
Internalizing	8.07 ± 6.35	12.81 ± 8.08	11.67 ± 9.79	.029	–	–
Externalizing	7.91 ± 6.30	13.74 ± 12.21	14.73 ± 13.67	.017	–	–
Dysregulation Profile	20.60 ± 9.09	24.96 ± 10.88	25.47 ± 16.48	.172	–	–

### Symptom ratings

Compared with the HR‐MAS group, the LR‐MAS group exhibited significantly greater baseline to endpoint reductions in ADHD‐RS inattention subscale scores (group‐by‐time interaction: *p* < .001) but not hyperactivity/impulsivity subscale scores (*p* = .585), and there was a trend for ADHD‐RS total score (*p* = .063) (Table S1). Remission rates (endpoint ADHD‐RS total score ≤ 18) for the LR‐MAS and HR‐MAS groups were 92.5% and 73.9%, respectively (*p* = .063). There were no significant group‐by‐time interaction effects for other symptom ratings between the HR‐MAS and LR‐MAS groups, and there were no significant group‐by‐time interactions for any symptom rating between the HR‐MAS and HR‐PLA groups.

### LR‐MAS vs. HR‐MAS

**Figure 1:**
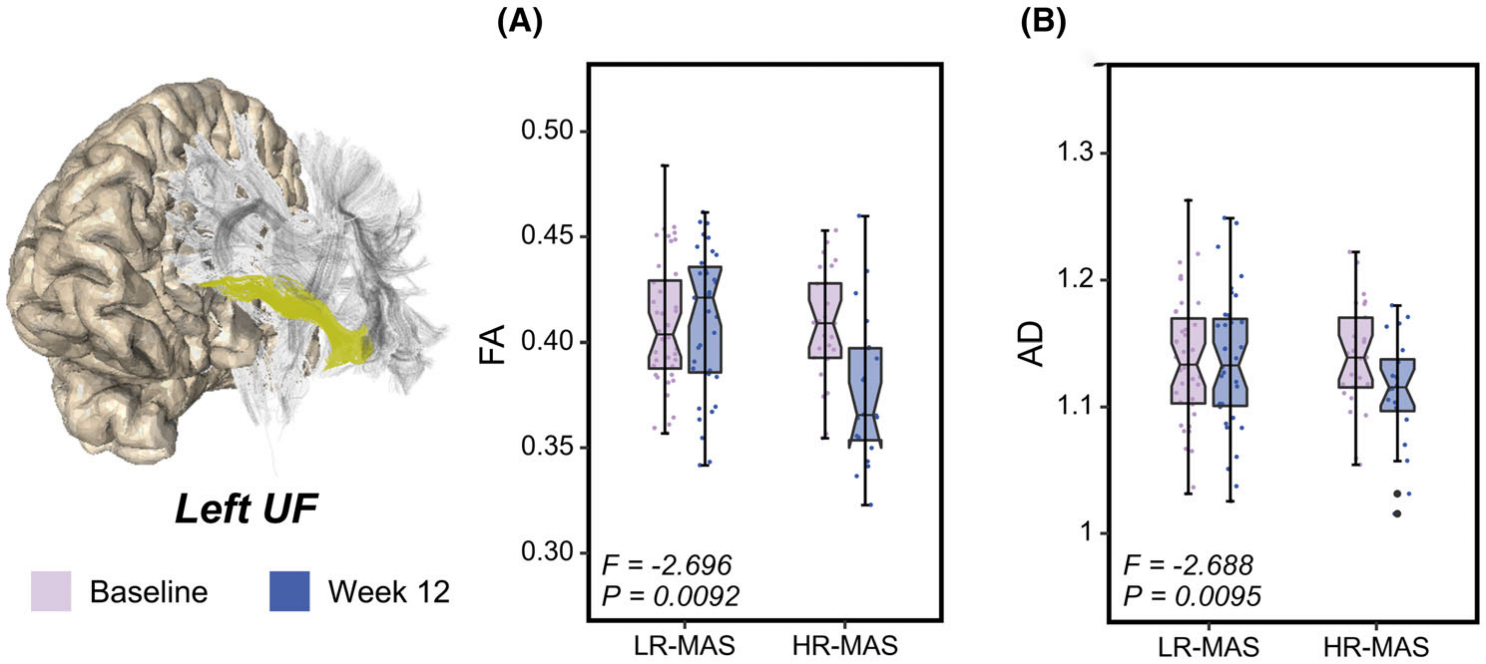
Differential baseline to week 12 changes in left UF fractional anisotropy (FA) (A) and axial diffusivity (AD) (B) in LR‐MAS and HR‐MAS groups following psychostimulant treatment

Significant group‐by‐time interactions between the LR‐MAS and HR‐MAS groups were observed for left UF FA (*p* = .0092) and UF AD (*p* = .0095) (Figure 1). There were no significant group‐by‐time interactions for any head motion parameter of translation, rotation, and framewise displacement (all *p* > .05; Table S2). Effect sizes for significant findings are presented in Table S3. In our sensitivity analyses, these results remained significant when controlling for pubertal status and head motion (Table S4). For the post hoc analysis, we did not find significant between‐group differences for left UF FA and UF AD at baseline. Following 12 weeks of MAS‐XR treatment, the HR‐MAS group exhibited significant reductions in the left UF FA (*p* = .0028) and UF AD (*p* = .0061), whereas no significant changes were observed in the LR‐MAS group. At week 12, left UF FA was significantly lower in the HR‐MAS relative to the LR‐MAS group (*p* = .0005).

**Figure 2:**
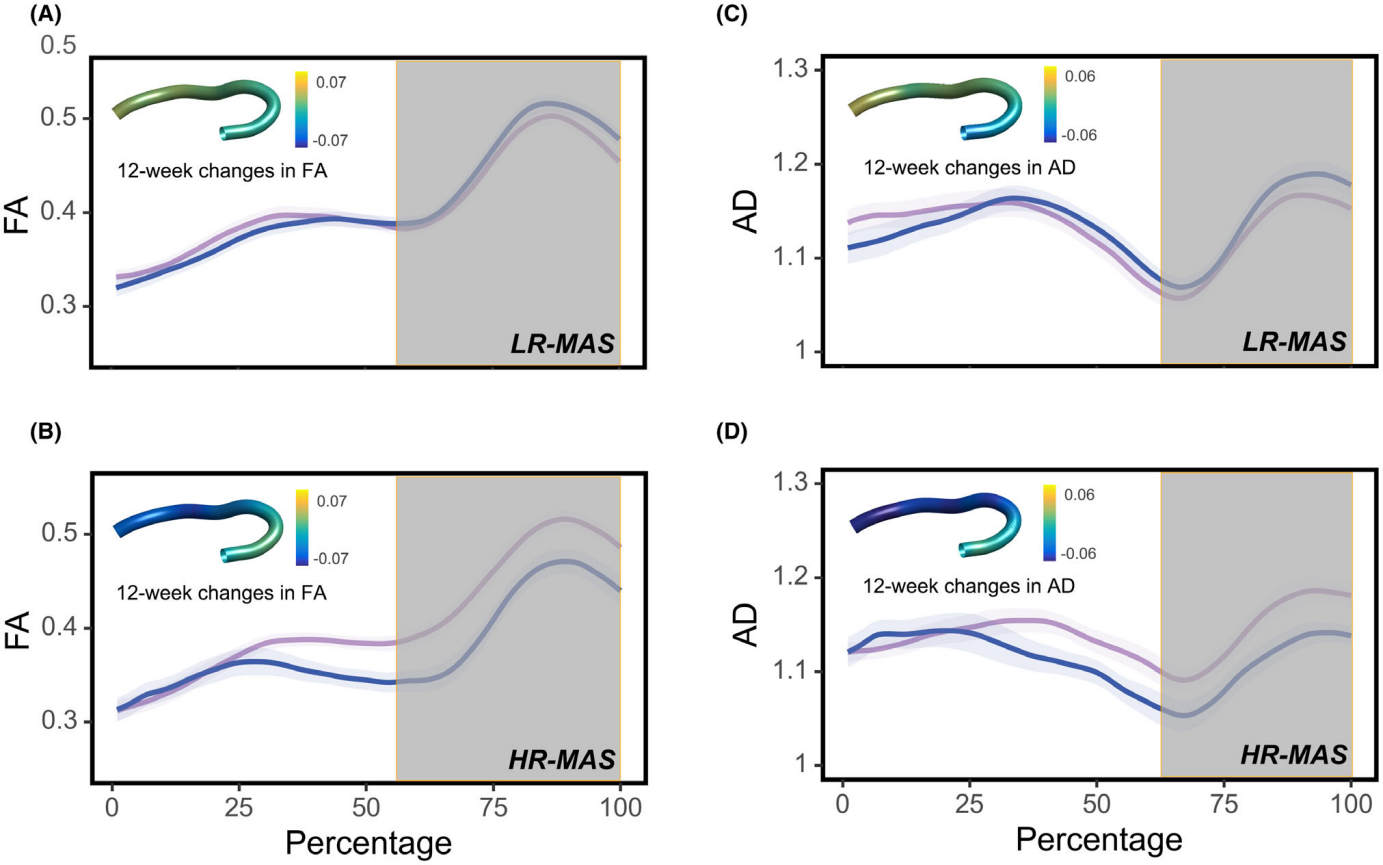
Differential baseline to week 12 changes in fractional anisotropy (FA) (A, B) and axial diffusivity (AD) (C, D) along the length of the left UF fiber tract within LR‐MAS and HR‐MAS groups following psychostimulant treatment. Segments with significant group‐by‐time interaction effects are shown in gray shades

For the segment‐level comparison, significant group‐by‐time interaction effects were identified in the anterior portion of the left UF for FA (segments 67–100) and AD (segments 82–100) (Figure 2). The post hoc within‐group analyses of time effects revealed that the HR‐MAS but not LR‐MAS group exhibited a significant decrease in FA and AD of the anterior segment of the left UF. In addition, the HR‐MAS group exhibited significantly lower FA and AD in the anterior segment of the left UF relative to the LR‐MAS group at week 12, whereas no differences were observed at baseline.

### Secondary contrasts

Significant group‐by‐time interactions were observed for left UF FA (*p* = .0010) and UF AD (*p* = .0487) in the HR‐MAS but not LR‐MAS group when compared with HC (Figure S3). These results remained significant when controlling for pubertal status and head motion (Table S4). For the post hoc simple effect analysis, no baseline between‐group differences were observed for the left UF FA or UF AD. The HC did not exhibit significant changes over 12 weeks of MAS‐XR treatment, whereas HR‐MAS, but not LR‐MAS, exhibited a significant reduction in the left UF FA (*p* = .0017) and UF AD (*p* = .0020). At week 12, the HR‐MAS group exhibited significantly lower left UF FA (*p* = .0021) and UF AD (*p* = .0032) relative to HC.

When comparing HR‐MAS and HR‐PLA groups, a significant group‐by‐time interaction was observed for left UF FA (*p* = .044) but not AD (*p* = .696) (Figure S3). These results remained significant when controlling for pubertal status and head motion (Table S4). Specifically, 12 weeks of MAS‐XR treatment significantly reduced the left UF FA in the HR‐MAS (*p* = .0088) but not the HR‐PLA group (*p* = .66). There were no significant between‐group differences at baseline and week 12. There were no significant group‐by‐time interactions for left UF FA (*p* = .69) and left UF AD (*p* = .42) in HR‐PLA relative to HC.

### Correlation between changes in the UF diffusion metrics and symptom ratings

After correcting for multiple comparisons, there were no significant correlations between changes in left UF FA or AD and clinical ratings within and among both LR‐MAS and HR‐MAS groups (Figure S4).

### Whole‐brain exploratory findings

No significant group‐by‐time interaction effects were observed among the remaining major fiber tracts reconstructed by AFQ (all *p* > .05). For the TBSS analysis, we did not find any clusters showing significant differences in baseline‐endpoint microstructural changes between the LR‐MAS and HR‐MAS groups.

## Discussion

This study investigated psychostimulant‐related UF white matter microstructural changes in ADHD youth at varying levels of BD familial risk. Contrary to our first hypotheses, at baseline, HR‐ADHD youth did not exhibit UF FA deficits compared with either HC or LR‐ADHD youth. In support of our second hypothesis, following 12 weeks of psychostimulant treatment, significant group‐by‐time interactions were found in left UF FA and UF AD between LR‐MAS and HR‐MAS groups. Specifically, the HR‐MAS group exhibited a significant decrease in the left UF FA and UF AD, whereas the LR‐MAS group did not. Additionally, reductions in these diffusion metrics were not exhibited by either HC or HR‐PLA groups. At the segment level, we further revealed that such treatment‐induced FA and AD reductions were primarily localized in the anterior prefrontal portion of the left UF. Symptom reductions were generally greater in the LR‐MAS compared to the HR‐MAS group, particularly for ADHD‐RS inattention subscale scores, though these clinical changes showed no significant correlation with alterations in left UF FA or AD. Symptom improvements in the HR‐PLA group are generally consistent with the well‐documented placebo response commonly observed in ADHD trials (Faraone et al., 2022). Notably, the comparable symptom reductions between the HR‐MAS and HR‐PLA groups might suggest diminished MAS effectiveness in HR‐ADHD youth. Together, these findings demonstrate that neither HR‐ADHD nor LR‐ADHD youth exhibit abnormalities in bilateral UF microstructural integrity prior to psychostimulant treatment and that HR‐ADHD youth uniquely exhibit reductions in the left UF microstructural integrity following 12 weeks of psychostimulant treatment.

Based on prior evidence that youth with BD exhibit reduced UF integrity relative to HC (Xu et al., 2022), we hypothesized that youth with both BD family history and ADHD, two risk factors for BD, would also exhibit UF microstructural deficits. Contrary to our hypothesis, however, there were no UF microstructural deficits in HR‐ADHD youth relative to HC at baseline. This finding is consistent with a recent meta‐analysis finding that UF microstructural integrity is not reduced in individuals at familial risk for BD (Xu et al., 2022). It is notable that most DTI findings of reduced UF FA in high‐risk individuals were based on adults (Foley et al., 2018; Linke et al., 2013), and longitudinal studies have revealed distinct UF microstructural trajectories between BD and HC during late adolescence and early adulthood (Weathers et al., 2018). Therefore, aberrant UF microstructure appears to be closely related to neurodevelopment, and UF integrity deficits in individuals at risk for BD may progressively decline after adolescence. In addition, we found that LR‐ADHD youth did not exhibit abnormal UF FA or UF AD compared with HC, which is consistent with prior meta‐analytic evidence in ADHD youth (Aoki et al., 2018; Zhang et al., 2023). Collectively, these findings suggest that neither ADHD nor genetic vulnerability to BD significantly alters normal UF FA and UF AD developmental trajectories in youth.

A primary finding of our study is that 12 weeks of psychostimulant treatment uniquely decreased left UF FA in HR‐ADHD but not LR‐ADHD youth. Importantly, the reduction in left UF FA was not observed in the HR placebo group, suggesting a pharmacological effect rather than a progressive decline in HR‐ADHD youth over time. While the mediating pharmacological mechanisms are not known, it may be relevant that rodent studies have shown that elevated dopamine neurotransmission increases excitatory prefrontal‐amygdala functional connectivity (Floresco & Tse, 2007) and that repeated stimulant administration uniquely increases neuronal excitability and dendritic atrophy in the orbitofrontal cortex (Crombag, Gorny, Li, Kolb, & Robinson, 2005; Homayoun & Moghaddam, 2006). Notably, HR‐ADHD youth also exhibited reductions in left UF AD, a diffusivity measure parallel and perpendicular to the fiber tracts (Song et al., 2003), and may indicate that UF microstructural changes in response to psychostimulants were related to reductions in axonal rather than myelin integrity. In addition, the observation that LR‐ADHD youth did not exhibit UF microstructural alterations following 12‐week psychostimulant treatment is consistent with prior cross‐sectional evidence for similar UF FA levels in stimulant‐naïve and stimulant‐treated ADHD youth (de Luis‐García et al., 2015; Schweren et al., 2013), and a controlled trial finding that 16‐week methylphenidate treatment did not significantly alter UF FA in initially stimulant‐naïve ADHD boys (Bouziane et al., 2019). Together, these findings suggest that HR‐ADHD youth are uniquely vulnerable to UF microstructural degradation in response to psychostimulants, and additional studies are warranted to clarify potential mediating heritable genetic and/or epigenetic mechanisms.

It is also notable that psychostimulant treatment selectively reduced FA of the left but not right UF in HR‐ADHD youth. While the reasons underlying this lateralized effect are not known, it may be relevant that meta‐analytic evidence has implicated left‐lateralized prefrontal‐amygdala circuit involvement in emotional processing (Baas, Aleman, & Kahn, 2004; Berboth & Morawetz, 2021). Furthermore, we previously reported that psychostimulant‐free HR‐ADHD youth exhibit left‐lateralized ventrolateral prefrontal (VLPFC) and amygdala hyperactivation in response to emotional stimuli (Patino et al., 2024). Additionally, we found that the reduction in left UF FA was mainly restricted to the anterior segment localized in the VLPFC and orbitofrontal cortex, which play an integral role in social and emotional processing. Interestingly, previous studies consistently identified lower FA in the orbitofrontal cortex segment of the UF in BD patients (Deng et al., 2018; Versace et al., 2010). Together, these findings suggest that the UF segment localized in the left VLPFC and orbitofrontal cortex is most vulnerable to psychostimulant‐induced degradation in HR‐ADHD youth, potentially due to prefrontal–amygdala circuit structural and functional changes.

Unexpectedly, we found no significant correlations between changes in left UF microstructural metrics and clinical ratings within or among both LR‐MAS and HR‐MAS groups. It is possible that our sample sizes were not sufficient to detect potential relationships with small to moderate effect sizes. Given the well‐established evidence of UF microstructural deficits in BD patients (Xu et al., 2022), we had anticipated that the observed decline in left UF FA would correlate with corresponding more severe mood symptoms. However, reductions in manic and depressive symptom severity were instead observed. We speculate that compensatory brain functional activities may be involved and mask potential relationships between UF deficits and mood changes. Moreover, a prior study has linked lower UF integrity to mania progression in non‐BD individuals over 6‐month follow‐up periods (Santos et al., 2022). A 6‐year prospective study found that lower UF FA significantly predicted the onset of BD in offspring of parents with BD (Li et al., 2021). These findings suggest that longer observation periods (>12 weeks) may be necessary to detect significant correlations between psychostimulant‐induced UF microstructural impairment and mood symptom alterations.

This study has several limitations. First, the duration of psychostimulant treatment was relatively short, and more robust or different changes in white matter may emerge following longer psychostimulant exposure. Second, the sample size was relatively small, which may have limited our ability to detect more subtle changes. Third, the diffusion‐weighted imaging acquisition only included a single b value, and multi‐shell diffusion imaging in conjunction with advanced diffusion models may provide a more robust estimation of diffusion metrics. Fourth, detailed clinical information about BD index cases was not systematically collected to evaluate the potential impact on the current findings. Strengths of this study include a well‐characterized cohort of psychostimulant‐free ADHD youth with and without BD family history with similar demographics, inclusion of a healthy comparison group, uniform psychostimulant monotherapy, and prospective placebo‐controlled study design.

In conclusion, to our knowledge, this is the first study to systematically investigate UF microstructural integrity in ADHD youth with and without a BD family history prior to and following psychostimulant exposure. The results demonstrate that neither HR‐ADHD nor LR‐ADHD youth exhibited UF microstructural abnormalities prior to psychostimulant treatment. However, 12‐week psychostimulant treatment uniquely decreases left UF microstructural integrity, primarily localized to the anterior segment, in HR‐ADHD youth but not LR‐ADHD or placebo‐treated HR‐ADHD youth. While additional studies are warranted to clarify specific mechanisms mediating these effects, this finding suggests that to‐be‐defined heritable genetic and/or epigenetic factors may increase UF vulnerability to degradation in response to psychostimulants in HR‐ADHD youth. In view of prior prospective evidence that lower UF FA is associated with increased risk for developing BD and that BD patients exhibit bilateral UF FA deficits, additional studies are warranted to investigate the relationship between long‐term naturalistic psychostimulant treatment and BD risk progression in HR‐ADHD youth.

## Ethical considerations

Written informed assent and consent were obtained from youth and their legal guardians, respectively. This study was approved by the University of Cincinnati Institutional Review Board (#2015‐2726; date: 05/16/2018).

## Trial registration

This study was registered at ClinicalTrials.gov (NCT02478788) in June 2015. Enrollment took place from October 2015 to December 2021, and the DTI analyses were initiated in May 2022.

**Key points**

**What's known?**

- Psychostimulants are a first‐line treatment for ADHD irrespective of familial risk for bipolar disorder (BD) and may accelerate BD risk progression. Therefore, it is important to clarify the impact of psychostimulant exposure on neural systems implicated the pathophysiology of BD in this high‐risk population.

**What's new?**

- This is the first study to systematically compare the effects of twelve week psychostimulant treatment on white matter microstructural integrity in ADHD youth with and without a BD familiy history. We found that psychostimulant treatment uniquely decreased microstructural integrity of the left uncinate fasciculus in ADHD youth with but not without a BD familiy history.

**What's relevant?**

- Our findings highlight an adverse effect of psychostimulants in ADHD youth with familial risk for BD suggesting that alternate treatment strategies are needed for this high‐risk population.

## Supporting information


**Table S1.** Differential baseline‐endpoint changes in clinical ratings among ADHD youth.
**Table S2**. Group‐by‐time interaction effects on all head motion parameters.
**Table S3**. Statistical summary of significant group‐by‐time interaction effects on DTI metrics.
**Table S4**. Sensitivity analyses results.
**Figure S1**. Overview of the clinical trial design.
**Figure S2**. (A) CONSORT diagram for ADHD patients. (B) CONSORT diagram for healthy control subjects.
**Figure S3**. Secondary analyses of 12‐week changes in the left UF fractional anisotropy (FA) and axial diffusivity (AD) in LR‐MAS and HR‐MAS groups following psychostimulant treatment relative the healthy controls (HC), and in the HR‐MAS group relative to HR‐PLA.
**Figure S4**. Heat maps illustrating correlation coefficients (top) and significance levels (bottom) for associations between baseline‐week 12 changes in clinical ratings and left UF fractional anisotropy (FA) and axial diffusivity (AD) within and among both LR‐MAS and HR‐MAS groups.

## Data Availability

Data used in this study are available upon reasonable request and application.
