# Supplementary material for: Psychostimulant treatment uniquely reduces left uncinate fasciculus microstructural integrity in ADHD youth with a familial risk for bipolar I disorder: a 12‐week DTI study
Source: J Child Psychol Psychiatry. 2025 Sep 17;67(5):696–706. doi: 10.1111/jcpp.70053 (PMC13102043; doi:10.1111/jcpp.70053)

**Supplemental Information**

**1. Subject attrition and data quality control**

A total of 152 youth who met the inclusion and exclusion criteria were enrolled in this study (HR-PLA:18; HR-MAS:35; LR-MAS:51; HC:48). During the 12-week trial, 28 youth were lost due to one or more reasons including the following: a) lost to follow-up (HR-PLA:2; HR-MAS:7; LR-MAS:4; HC:7), b) adverse event (HR-MAS:2), c) Lack of efficacy (HR-PLA:1; HR-MAS:1), d) consent withdrawn (HR-PLA:2; HR-MAS:1), e) Schedule conflict (HR-MAS:1). A total of 126 youth (HR-PLA:13; HR-MAS:23; LR-MAS:47; HC:41) completed the 12-week trial.

There were 138 youth (HR-PLA:16; HR-MAS:29; LR-MAS:48; HC:45) who completed the baseline DTI scans. One baseline scan was discarded because of excessive head motion (LR-MAS:1). A total of 137 baseline scans (HR-PLA:16; HR-MAS: 29; LR-MAS:47; HC:45) were included in our analysis. A total of 112 youth (HR-PLA:11; HR-MAS:23; LR-MAS:41; HC:37) finished the week-12 MRI scans. one week-12 scans were excluded for excessive head motion (LR-MAS:1). A total of 111 week-12 scans (HR-PLA:11; HR-MAS:23; LR-MAS:40; HC:37) were included in the final analyses.

**2. Whole-brain exploratory analyses**

To explore potential changes throughout the whole-brain, group-by-time interaction effects between LR-MAS and HR-MAS groups were performed at tract- and voxel-level separately. For the tract-level analysis, we used AFQ pipeline and focused on the remaining 18 major fiber tracts throughout the whole brain in addition to bilateral UF. Linear mixed effects model was applied with age and sex as covariates. The FDR correction was used for multiple comparison issues. Moreover, we adopted TBSS to perform voxel-level whole-brain microstructural analysis. FA data from each participant were further processed and analyzed using the Tract-Based Spatial Statistic (TBSS) tool available in FSL. The TBSS procedure was as follows. FA images were normalized to the standard FMRIB58 FA template using the nonlinear registration algorithm in FSL. The normalized FA images were averaged to create the mean FA map. The mean FA map was used to generate the FA skeleton, with a threshold of 0.2 to exclude voxels that were primarily gray matter or cerebrospinal fluid. After FA skeleton generation, each individual subject’s FA was projected onto the FA skeleton. We implemented linear mixed model with 3dLME function in AFNI to test for FA differences between groups. White matter (WM) abnormalities were investigated using the FWE corrected P value <0.05. A similar tract-based analysis procedure was also applied to the MD, AD and RD images.

**Table S1** Differential baseline-endpoint changes in clinical ratings among ADHD youth.

| **Clinical Ratings** | **LR-MAS** | **HR-MAS** | **HR-PLA** | ***P* value ^a^**  **(LR-MAS vs. HR-MAS)** | ***P* value ^b^**  **(HR-MAS vs. HR-PLA)** |
| --- | --- | --- | --- | --- | --- |
| ***ADHD-RS total score*** | | | | | |
| Baseline | 33.17±9.83 | 34.97±9.51 | 35.56±12.99 | 0.063 | 0.305 |
| Endpoint | 7.85±8.52 | 13.91±10.94 | 20.00±9.23 |  |  |
| ***ADHD-RS inattention score*** | | | | | |
| Baseline | 20.83±4.83 | 19.59±5.14 | 19.19±6.75 | **<0.001** | 0.123 |
| Endpoint | 4.30±4.46 | 8.43±6.44 | 12.18±6.71 |  |  |
| ***ADHD-RS hyperactivity/impulsivity score*** | | | | | |
| Baseline | 12.34±7.94 | 15.38±7.22 | 16.38±7.71 | 0.585 | 0.812 |
| Endpoint | 3.55±4.53 | 5.48±5.67 | 7.82±4.56 |  |  |
| ***CDRS-R score*** | | | | | |
| Baseline | 24.06±5.96 | 27.72±8.59 | 27.19±8.83 | 0.670 | 0.664 |
| Endpoint | 20.48±3.04 | 24.09±8.85 | 24.36±8.08 |  |  |
| ***YMRS score*** | | | | | |
| Baseline | 3.06±3.32 | 5.31±4.20 | 6.75±8.09 | 0.277 | 0.270 |
| Endpoint | 1.10±2.04 | 2.17±2.33 | 1.36±2.01 |  |  |
| ***CBCL total score*** | | | | | |
| Baseline | 36.60±17.22 | 52.67±24.72 | 50.80±33.16 | 0.874 | 0.969 |
| Endpoint | 16.64±14.44 | 31.10±23.63 | 32.91±31.04 |  |  |
| ***CBCL internalizing score*** | | | | | |
| Baseline | 8.07±6.35 | 12.81±8.08 | 11.67±9.79 | 0.557 | 0.584 |
| Endpoint | 3.92±4.20 | 7.52±6.35 | 6.27±6.71 |  |  |
| ***CBCL externalizing score*** | | | | | |
| Baseline | 7.91±6.30 | 13.74±12.21 | 14.73±13.67 | 0.420 | 0.473 |
| Endpoint | 3.22±4.28 | 8.67±10.88 | 8.64±11.66 |  |  |
| ***CBCL dysregulation profile score*** | | | | | |
| Baseline | 20.60±9.09 | 24.96±10.88 | 25.47±16.48 | 0.147 | 0.676 |
| Endpoint | 9.00±7.48 | 15.62±11.26 | 16.36±13.22 |  |  |

^a^ P values for the group-by-time interaction effects between LR-MAS and HR-MAS.

^b^ P values for the group-by-time interaction effects between HR-MAS and HR-PLA.

*Abbreviations*: ADHD-RS, Attention-deficit/Hyperactivity Disorder Rating Scale; CDRS-R, Children’s Depression Rating Scale-Revised; YMRS, Young Mania Rating Scale; CBCL, Children Behavior Checklist.

**Table S2** Group-by-time interaction effects on all head motion parameters.

| **Contrasts** | ***P* values** | | | | | | |
| --- | --- | --- | --- | --- | --- | --- | --- |
|  | **FD** | **Translation (X)** | **Translation (Y)** | **Translation (Z)** | **Rotation**  **(X)** | **Rotation**  **(Y)** | **Rotation**  **(Z)** |
| LR-MAS vs. HR-MAS | 0.401 | 0.455 | 0.391 | 0.750 | 0.192 | 0.435 | 0.794 |
| LR-MAS vs. HC | 0.796 | 0.052 | 0.188 | 0.390 | 0.234 | 0.785 | 0.358 |
| HR-MAS vs. HC | 0.278 | 0.208 | 0.649 | 0.612 | 0.880 | 0.356 | 0.467 |
| HR-MAS vs. HR-PLA | 0.557 | 0.698 | 0.178 | 0.457 | 0.705 | 0.122 | 0.635 |

*Abbreviations:* FD, framewise displacement*.*

**Table S3** Statistical summary of significant group-by-time interaction effects on DTI metrics.

| **Response Variable** | **β_int_** | **95% CI** | **Cohen’s d** | **Marginal R^2^** | **Conditional R^2^** | ***P* value** |
| --- | --- | --- | --- | --- | --- | --- |
| ***LR-MAS vs. HR-MAS*** | | | | | | |
| Left UF-FA | -0.0317 | [-0.0549, -0.0084] | -1.0227 | 0.1438 | 0.1446 | 0.0092 |
| Left UF-AD | -0.0338 | [-0.0593, -0.0089] | -0.6609 | 0.0550 | 0.6650 | 0.0095 |
| ***HR-MAS vs. HC*** | | | | | | |
| Left UF-FA | -0.0416 | [-0.0652, -0.0179] | -1.2595 | 0.1609 | 0.3848 | 0.0010 |
| Left UF-AD | -0.0240 | [-0.0477, -0.0003] | -0.5366 | 0.1522 | 0.7077 | 0.0487 |
| ***HR-MAS vs. HR-PLA*** | | | | | | |
| Left UF-FA | 0.0354 | [0.0011, 0.0697] | 1.0797 | 0.1613 | 0.1613 | 0.0438 |

*Abbreviations:* FD, framewise displacement; UF, uncinate fasciculus; FA, fractional anisotropy; AD, axial diffusivity.

**Table S4.** Sensitivity analyses results

| **Metrics** | **LR-MAS vs. HR-MAS** | | **LR-MAS vs. HC** | | **HR-MAS vs. HC** | | **HR-MAS vs. HR-PLA** | |
| --- | --- | --- | --- | --- | --- | --- | --- | --- |
|  | ***F*** | ***P*** | ***F*** | ***P*** | ***F*** | ***P*** | ***F*** | ***P*** |
| ***Main results*** | | | | | | | | |
| Left UF-FA | -2.696 | **0.0092** | -0.847 | 0.4007 | -3.513 | **0.0010** | 2.054 | **0.0438** |
| Left UF-AD | -2.688 | **0.0095** | 0.729 | 0.4686 | -2.030 | **0.0487** | 0.394 | 0.6955 |
| ***Including absolute FD as covariate*** | | | | | |  |  |  |
| Left UF-FA | -2.695 | **0.0092** | -0.774 | 0.4422 | -3.516 | **0.0009** | 2.171 | **0.0334** |
| Left UF-AD | -2.696 | **0.0094** | 0.686 | 0.4952 | -2.030 | **0.0487** | 0.394 | 0.6959 |
| ***Including pubertal status*** ***as covariate*** | | | | | | | | |
| Left UF-FA | -2.911 | **0.0043** | -0.895 | 0.3744 | -3.573 | **0.0008** | 2.069 | **0.0423** |
| Left UF-AD | -2.600 | **0.0120** | 0.716 | 0.4771 | -2.066 | **0.0451** | 0.331 | 0.7420 |

*Abbreviations:* FD, framewise displacement; UF, uncinate fasciculus; FA, fractional anisotropy; AD, axial diffusivity.

**Figure S1.** Overview of the clinical trial design


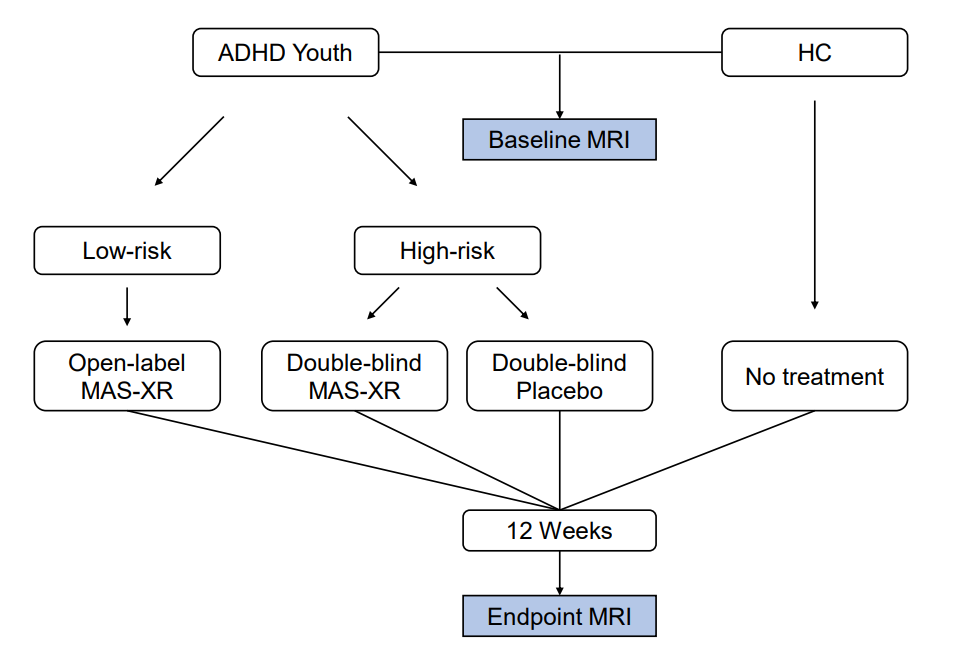

**Figure S2A.** CONSORT Diagram for ADHD patients

**Figure S2B.** CONSORT Diagram for Healthy Control Subjects

**Figure S3.** Secondary analyses of 12-week changes in the left UF fractional anisotropy (FA) and axial diffusivity (AD) in LR-MAS and HR-MAS groups following psychostimulant treatment relative the healthy controls (HC), and in the HR-MAS group relative to HR-PLA.


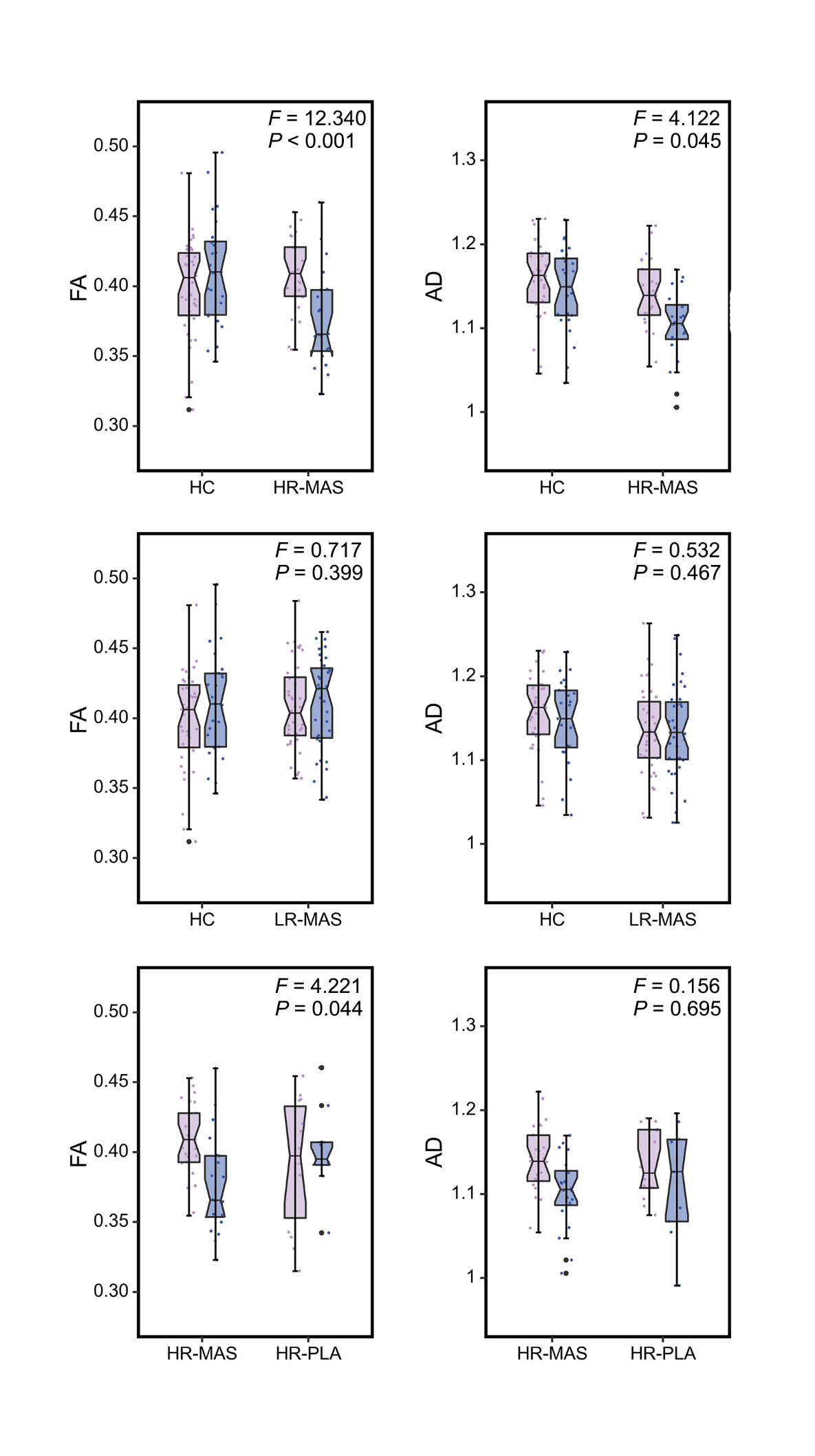


**Figure S4.** Heat maps illustrating correlation coefficients (top) and significance levels (bottom) for associations between baseline-week 12 changes in clinical ratings and left UF fractional anisotropy (FA) and axial diffusivity (AD) within and among both LR-MAS and HR-MAS groups.
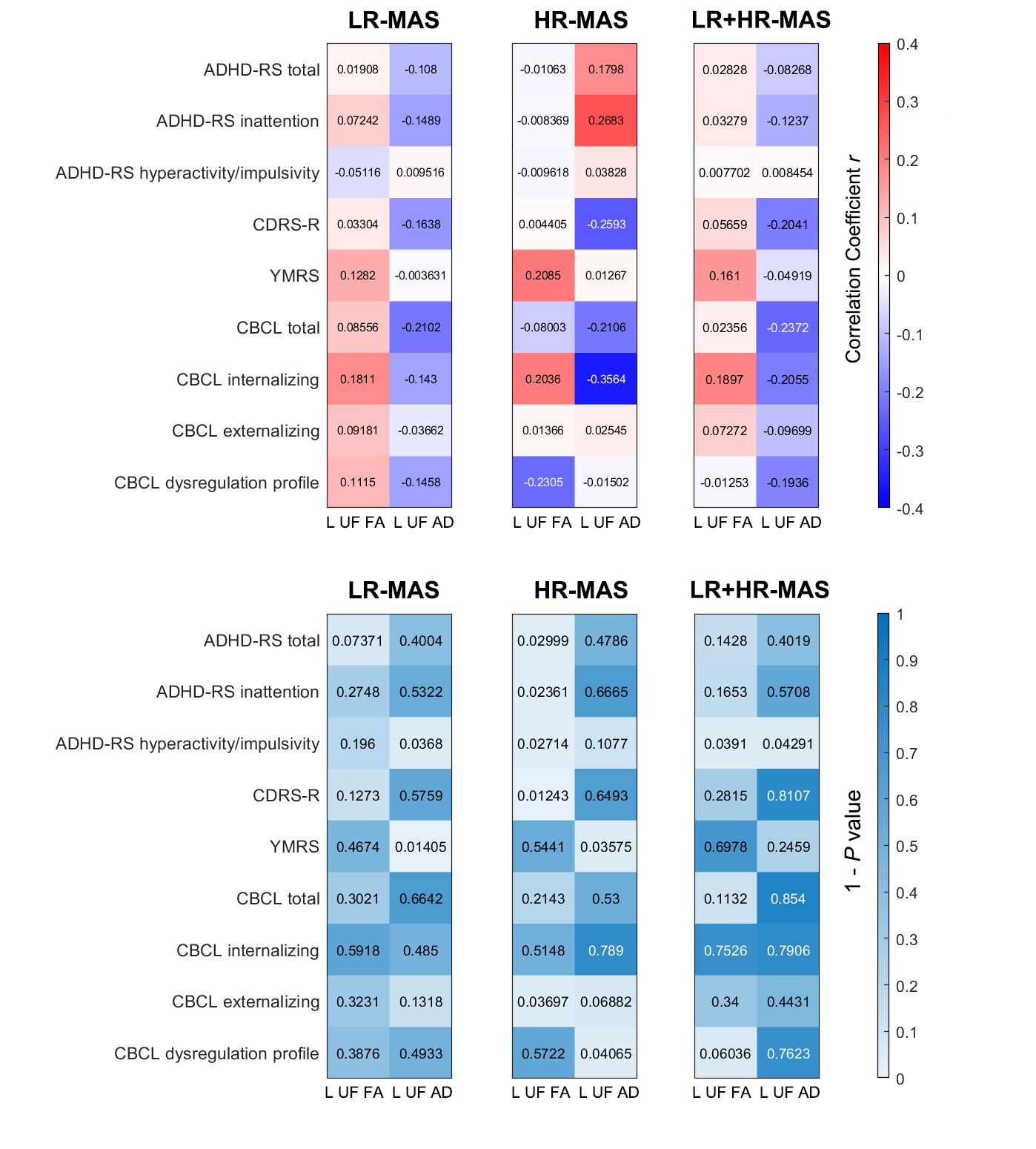

Supplement: Supplementary file 1 — Table S1. Differential baseline‐endpoint changes in clinical ratings among ADHD youth. Table S2. Group‐by‐time interaction effects on all head motion parameters. Table S3. Statistical summary of significant group‐by‐time interaction effects on DTI metrics. Table S4. Sensitivity analyses results. Figure S1. Overview of the clinical trial design. Figure S2. (A) CONSORT diagram for ADHD patients. (B) CONSORT diagram for healthy control subjects. Figure S3. Secondary analyses of 12‐week changes in the left UF fractional anisotropy (FA) and axial diffusivity (AD) in LR‐MAS and HR‐MAS groups following psychostimulant treatment relative the healthy controls (HC), and in the HR‐MAS group relative to HR‐PLA. Figure S4. Heat maps illustrating correlation coefficients (top) and significance levels (bottom) for associations between baseline‐week 12 changes in clinical ratings and left UF fractional anisotropy (FA) and axial diffusivity (AD) within and among both LR‐MAS and HR‐MAS groups. [file JCPP-67-696-s001.docx]
